# Elevated survivin expression in peripheral blood mononuclear cells is central to collateral formation in coronary chronic total occlusion

**DOI:** 10.3892/ijmm.2015.2154

**Published:** 2015-03-24

**Authors:** YIGUAN XU, XUERUI TAN, DONGMING WANG, WEI WANG, YUGUANG LI, MIN WU, SONGMING CHEN, YINGE WU, CHUNJIANG TAN

**Affiliations:** Department of Cardiology, The First Affiliated Hospital of Shantou University Medical College, Shantou, Guangdong 515041, P.R. China

**Keywords:** collateral vessel, survivin, peripheral blood mononuclear cells, vascular endothelial growth factor, intercellular adhesion molecule-1

## Abstract

Survivin is essential to angiogenesis and revascularization, but its role in coronary collateral formation remains unclear. The role of survivin in peripheral blood mononuclear cells (PBMCs) of coronary chronic total occlusion (CTO) patients was investigated. Coronary CTO patients (n=46; mean age 60.1±8.5, male 54.3%) (CTO group) and normal control patients (n=18; mean age 58.0±10.0, male 55.6%) underwent angiographic collateral vessel grading by Rentrop classification (C0 – C3) and provided peripheral blood between June 2006 and February 2007. Rat hind limb ischemia models were constructed using four equal groups of Sprague-Dawley rats (n=36): normal control, sham operation, operation and granulocyte macrophage colony-stimulating factor (GM-CSF). PBMC numbers and characteristics, collateral vessels, survivin, CD4, CD8, CD44, vascular endothelial growth factor (VEGF) and intercellular adhesion molecule-1 (ICAM-1) expression were determined using RT-PCR, flow cytometry, immunocytochemistry and western blot analysis. PBMC survivin mRNA and protein expression levels were higher in patients with good collateral circulation (C2 + C3) than in patients with no collateral flow (C0) (all P<0.05). Survivin single-positive and survivin and CD8, VEGF and ICAM-1 double-positive percentages were elevated in patients with good collateral circulation compared to those with normal and no collateral flow (all P<0.05), consistent with the rat model results, wherein higher survivin levels produced significantly larger and more visible collateral vessels. In conclusion, elevated survivin expression in PBMCs, particularly survivin and CD8, VEGF, and ICAM-1 double-positive PBMCs, may be crucial for good collateral formation in patients with coronary CTO, as confirmed by assessment of a rat model.

## Introduction

Cardiovascular diseases, in particular coronary artery disease (CAD), remain leading causes of mortality in developed countries (1). Coronary chronic total occlusion (CTO) affects up to 18.4% of all CAD patients and significantly influences cardiac function and outcomes of treatments, such as angioplasty and surgical bypass (2). Coronary collateral circulation (CCC) provides a natural bypass that supplies heart, potentially with therapeutic potential to improve myocardial viability in CAD patients (1,3). Survivin, a unique member of the inhibitor of apoptosis proteins (IAPs) family that exhibits cell cycle-regulated expression peaking at mitosis, has been demonstrated to impact cardiomyocyte replication and apoptosis, providing a potentially promising myocardial regeneration target (4). The role of survivin in peripheral blood mononuclear cells (PBMCs), cells whose gene expression profiles have been linked to CAD severity (5), remains unknown.

The role of survivin in angiogenesis is well documented, particularly in tumor cells (6). It has been demonstrated that survivin is minimally expressed in non-proliferating endothelial cells, but upregulated in newly formed blood vessels (7). Survivin, however, also plays a role in collateral vessel formation, as demonstrated by increasing microvessel density in survivin-positive tumors (8). In CAD patients, survivin influences cardiac function by controlling total cardiomyocyte numbers through its impact of collateral vessel formation and angiogenesis (4). Furthermore, Zwerts *et al* (9) reported that regulation of endothelial cell survival and maintenance of vascular integrity by survivin are crucial for normal embryonic angiogenesis, cardiogenesis and neurogenesis, demonstrating the importance of survivin in vascularization and revascularization.

In CTO patients, the role of CCC has been widely disputed; however, modern study has generally indicated that well-developed CCC is indicative of severe stenosis (10). When cardiac events occur, such as acute myocardial infarction, the presence of a well-developed CCC can mediate the detrimental effects of ischemia on heart tissues, thus preserving left ventricular function, reducing overall infarct size, preventing left ventricular aneurysm and increasing survival (10). Notably, collateral blood flow is often reduced after successful CTO recanalization, as antegrade blood flow is re-established and resistance is increased in collateral vessels (10). Thus, collateral vessel formation may be observed as a marker of stenosis and prognosis in CAD patients.

Altered survivin expression may impact collateral vessel formation, as indicated by Conway *et al* (11) who showed that survivin was uniquely expressed by microvessels in the peri-infarct and infarct regions 2 days after permanent artery occlusion. Furthermore, using a mouse model with heterozygous deficiency of middle cerebral of the survivin gene (survivin^+/‒^ mice), no alterations in infarct size were apparent (11). As the microRNA signature of PBMCs, including survivin, has been linked to CAD (5), it is likely that these cells also play a role in collateral formation. Furthermore, rising levels of vascular endothelial growth factor (VEGF), an angiogenic and vasoprotective molecule modulated primarily by inflammatory mediators, may also impact collateral formation in CAD patients, and intercellular adhesion molecule-1 (ICAM-1) may impact collateral formation and CAD onset (12,13), although the relationship between these molecules and survivin in PBMCs is unknown. Assessment of survivin levels as well as other molecules in PBMCs may thus be linked with collateral formation.

While the role of survivin in angiogenesis is well documented, much less is known about the distinct role survivin plays in collateral formation during coronary CTO. The present study examined the clinical relationship between PBMC survivin expression and coronary collateral formation in humans and the PBMC signatures associated with collateral formation. Correlations of survivin, VEGF and ICAM-1 expression were also examined in peripheral blood samples from human patients, and these correlations were confirmed in a rat model of hind limb ischemia. These experiments provided a basis for assessment of collateral formation based on PBMC survivin levels, potentially useful in revascularization therapies for CTO and CAD.

## Materials and methods

### Study design

A total of 46 coronary CTO patients (mean age 60.1±8.5, male 54.3%) (CTO group) and 18 patients with normal coronary artery vascularity (mean age 58.0±10.0, male 55.6%) (control group) were included in a prospective study between June 2006 and February 2007 at the Department of Cardiology of the the First Affiliated Hospital of Shantou University Medical College (China). In addition, an animal model was established using 36 male Sprague-Dawley rats aged 4–5 months and weighing 250–300 g. The rats were randomly divided into four equal groups of 9 rats each, including the normal control, sham operation, operation and granulocyte macrophage colony-stimulating factor (GM-CSF) treatment groups. The study protocol was approved by the Ethics Committee of Shantou University Medical College. Procedures involving experimental animals were performed in accordance with protocols approved by Institutional Guidelines for Care and Use of Laboratory Animals of Shantou University Medical College, in compliance with the Law of the People’s Republic of China on the Protection of Wildlife (2004). The patients provided written informed consent for participation.

### Patients

Included patients in the coronary CTO group (n=46; mean age 60.1±8.5, male 54.3%) exhibited i) angiographic studies showing coronary CTO occlusion (>99% occluded) for >2 weeks in any 3 major coronary artery branches, consistent with the diagnostic guidelines of Teeuwen *et al* (14); ii) measurable collateral flow according to Rentrop classification prior to reopening of the artery, as previously described (15). Patients that presented with other serious illness that potentially affected testing, such as acute myocardial infarction, cancer, pulmonary heart disease and severe renal insufficiency were excluded. Control subjects experienced chest pain and were suspected to have coronary heart disease, although normal coronary artery flow was confirmed by angiography for the patients included in the control group. The study protocol was approved by the First Affiliated Hospital of Shantou University Medical College, Shantou, and all the participants provided written informed consent.

### Sample collection

Angiography was performed using the Judkins technique via the femoral approach with 6F guiding catheters as previously described (16). The patients received a bolus of heparin (50 IU/kg), aspirin (100 mg/day), and clopidogrel (75 mg/day) for ≥3 day when percutaneous coronary intervention (PCI) was performed. Approximately 10 ml of peripheral blood was drawn with moderate suction in a 10 sec period using the femoral approach, and angioplasty was continued.

### Angiographic grading

Angiograms were assessed independently by two blinded investigators, and in case of disagreement, a consensus was obtained (through consultation). According to the Rentrop classification (15), the angiographically visible diameter of collateral connections was graded as: C0, no opacification; C1, filling of artery side branches by collateral vessels without visualization of the epicardial segments; C2, partial filling of epicardial segments by collateral vessels; and C3, complete filling of epicardial segments by collateral vessels. Rentrop C1 was regarded as poor collateral circulation, and Rentrop C2 and C3 were regarded as good collateral circulation.

### PBMC isolation

PBMCs were separated by standard density gradient centrifugation using the Ficoll-Paque method (Biochrom, Berlin, Germany), according to the manufacturer’s instructions. PBMCs (3×10^6^ cells/ml) were then cultured in buffered RPMI-1640 supplemented with 10% (v/v) heat-inactivated fetal bovine serum (both from Sigma, St. Louis, MO, USA).

### Flow cytometry

PBMCs were washed once with phosphate-buffered saline (PBS) solution, fixed in 4% paraformaldehyde at room temperature for 40 min, and incubated in 0.2% Triton X-100 (Sangon Corp., Shanghai, China) for 10 min and 5% calf serum at 4°C for 10 min. PBMCs were then incubated in PBS containing survivin (1:1,000; Novus Biologicals, Inc., Littleton, CO, USA), CD4 (1:100), CD8 (1:100), CD44 (1:100) (all from Boster Biotechnology Co., Wuhan, China), VEGF (1:200), and ICAM-1 (1:200; Santa Cruz Biotechnology, Inc., Dallas, TX, USA) antibody overnight at 4°C. After washing, the PBMCs were incubated in appropriate fluorescein isothiocyanate (FITC) (survivin) and/or Cy3 (CD4, CD8, CD44, VEGF and ICAM-1)-conjugated secondary antibodies. Flow cytometric analysis was conducted using a Coulter Epics XL flow cytometer (Beckman Coulter, Brea, CA, USA), and the percentage of positive cells was calculated.

### Immunocytochemistry

Survivin expression in PBMCs was detected by immunocytochemical staining using an Ultravision Detection System (Lab Vision Corporation, Newmarket Suffolk, UK), as previously described (17). Briefly, after blocking endogenous peroxidase activity, PBMCs were incubated for 1 h at room temperature with rabbit polyclonal antibody to survivin (1:1,000; Novus Biologicals). After washing, the cells were incubated for 1 h at room temperature with biotinylated goat anti-rabbit IgG (1:10,000; Santa Cruz Biotechnology, Inc., Brea, CA, USA). Nuclei were counter-stained with hematoxylin. Three replicates were performed for each sample.

### RT-PCR detection of survivin mRNA expression in PBMCs

Total RNA was isolated from PBMCs using TRIzol reagent (British Biocell International, Cardiff, UK). RNA purity was determined using absorbance at 260 and 280 nm (A260/280), and RNA integrity was verified by 1.5% agarose gel electrophoresis (Invitrogen, Grand Island, NY, USA). Total RNA was reverse-transcribed into cDNA using RevertAid ^7M^M-MuLV Reverse Transcriptase and oligo(dT) 18 primer (both from Thermo Scientific, Waltham, MA, USA). Approximately 436 bp of the human survivin region and 612 bp of the β-actin region were generated by RT-PCR using total RNA (2 *μ*g). Primer sequences for survivin were: forward, 5′-ATG GGT GCC CCG ACG TTG-3′ and reverse, 5′-AGA GGC CTC AAT CCA TGG-3′. Primer sequences for β-actin were: forward, 5′-CGC TGC GCT GGT CGT CGA CA-3′ and reverse, 5′-GTC ACG CAC GAT TTC CCG CT-3′. Reactions involved initial denaturation at 95°C for 3 min followed by 35 cycles at 94°C for 1 min, annealing at 61°C for 1 min, and extension at 72°C for 1 min and an additional cycle at 72°C for 10 min. β-actin was used as an internal control. RT-PCR products were subjected to 1.5% agarose gel electrophoresis, and the abundance of each mRNA was normalized to β-actin using Image Lab (Bio-Rad, Hercules, CA, USA). Relative mRNA expression was considered to be the optical density (OD) of survivin by the OD of β-actin (OD_survivin_/OD_β-actin_).

### Western blot analysis

Western blot analysis was performed as previously described (17). Total protein was isolated from PBMCs using TRIzol Reagent (British Biocell International). Proteins were quantified by a Bradford assay. Total protein (50 *μ*g) was separated on 12% sodium dodecyl sulfate-polyacrylamide gel (SDS-PAGE) gels, and electro-transferred to NC membranes (Millipore, Bedford, MA, USA). After non-specific binding sites were blocked with 5% skim milk for 1 h, the membranes were then incubated in a mixture of Tris-buffered saline and Tween-20 (TBS-T) containing survivin (1:1,000), CD4 (1:100), CD8 (1:100), CD44 (1:100), VEGF (1:200) and ICAM-1 (1:200) antibodies for 2 h at room temperature. After washing, the membrane was incubated in TBS-T containing the appropriate secondary anti-IgG antibodies (1:5,000; Santa Cruz Biotechnology, Inc.) at room temperature for 1 h. The target protein was detected using western blotting luminol reagent (Santa Cruz Biotechnology, Inc.), according to the manufacturer’s instructions. Results were visualized by autoradiography using X-Omat autoradiography film (Kodak, Rochester, NY, USA). For protein loading normalization, a similar procedure was performed using a monoclonal antibody against tubulin (1:1,000; Beyotime Institute of Biotechnology, Hangzhou, China) as an internal standard. Three replicates were performed for each sample.

### Rat model of hind limb ischemia

Using four groups of 9 rats each (normal control, sham operation, operation and GM-CSF treatment groups) a rat model of hind limb ischemia was constructed, as previously described (18). For operation and GM-CSF treatment groups, the right femoral artery of each rat was ligated directly distal to the inguinal ligament to achieve hind limb ischemia on the right side. Sham operations were performed similarly, but without femoral artery ligation.

The day after surgery, the mice in the treatment group were administered rHuGM-CSF (10 *μ*g/kg/day; Xiamen Amoytop Biotech Co., Xiamen, China) in 0.9% normal saline solution by subcutaneous daily for 5 days. The control, sham operation, and operation groups were administered the same volume of 0.9% normal saline solution. At day 28 after the surgery, the rats were sacrificed by overdose of injected anesthetic drugs (phenobarbital sodium), blood samples were taken by cardiac puncture from each specimen, and postmortem angiography was performed. PBMCs were isolated from blood samples, and the number of cells positive for survivin, CD4, CD8, CD44, VEGF and ICAM-1 were detected by flow cytometry.

### Postmortem angiography

Postmortem angiography was performed as previously described (19). Briefly, peripheral vascular tissues were fully expanded to better reveal collateral vessels and then perfused through the descending aorta with PBS containing adenosine (1 g/l) (Bio Basic Inc., Amherst, NY, USA) as a vasodilator for 3 min at physiological pressure. Vasculature was fixed with 2% paraformaldehyde in PBS for 5 min, flushed with PBS for 2 min, and infused with 76% meglumine diatrizoate and 10% gelatin (1:1) in PBS as a contrast reagent. The contrast reagent was solidified by immersing whole specimens in ice for 5 min. Angiograms were taken at two different angles, resulting in stereoscopic images that were used in 3-dimensional (3D) collateral growth analysis.

### Statistical analysis

Data were analyzed using SPSS version 13.0 for Windows (SPSS Inc., Chicago, IL, USA). Data were presented as the means ± standard deviations (SD). Differences between groups were analyzed by one-way ANOVA and Student-Newman-Keuls (SNK) Games-Howell tests. P<0.05 was considered statistically significant (P<0.05).

## Results

### Baseline demographic and clinical characteristics of experimental and control patients

Of the total 46 patients, no collateral flow was observed in 12 patients (C0), poor collateral circulation was observed in 18 patients (C1), and good collateral circulation was observed in 16 patients (11 C2 patients and 5 C3 patients). No significant differences in baseline demographic or clinical characteristics, including age, gender, cardiovascular risk factors, median duration of occlusion were observed among the coronary CTO and control group patients (P>0.05) (Table I). Notably, all the coronary CTO patients were currently using vasoactive and lipid-lowering drugs, although none of the patients in the control group were on similar medication. Heparin was administered immediately prior to angiography in the coronary CTO and control patients.

### Survivin expression in human PBMCs during coronary collateral formation

**Figure 1:**
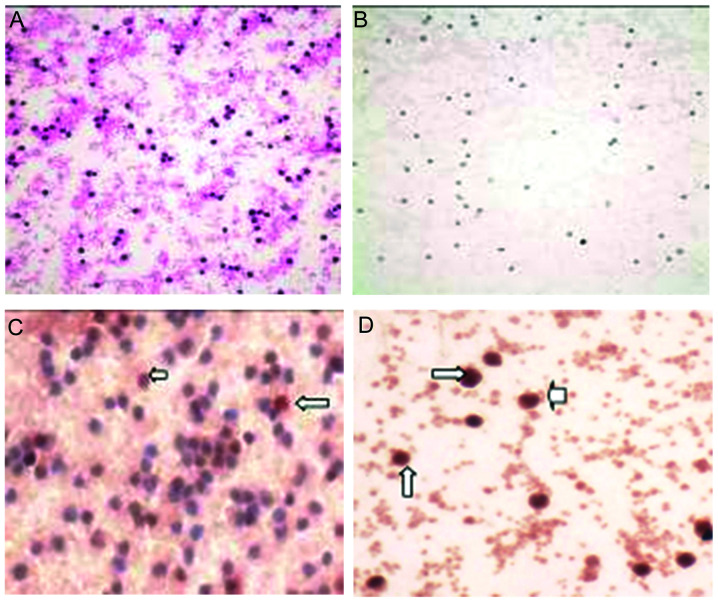
Immunocytochemical staining of human peripheral blood mononuclear cells (PBMCs) showing survivin expression (×400). (A) H&E staining of PBMCs showing clear and intact nuclei and plasma membranes in C2 patients. (B) C0 patients with no survivin-positive cells and without collateral formation. (C) Poor collateral formation (C1 patients) with survivin-positive reactions in nuclei and plasma membranes of some cells. (D) Good collateral formation (C2 + C3 patients) with survivin-positive reactions in the nuclei and plasma membranes. (C and D) Arrows indicate survivin-positive cells.

**Figure 2:**
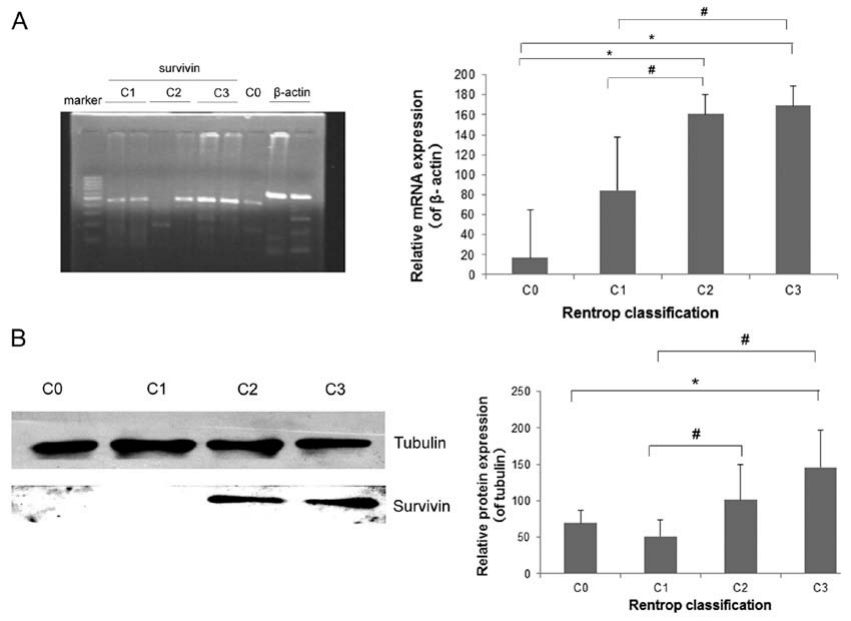
Survivin expression in human peripheral blood mononuclear cells (PBMCs) during coronary collateral formation. (A) Survivin mRNA expression in PBMCs during coronary collateral formation was determined by RT-PCR. β-actin was used as an internal control. Semi-quantitative analysis mRNA expression of survivin. (B) Survivin protein expression in PBMCs during coronary collateral formation was determined by western blotting. Tubulin was used as an internal control. Semi-quantitative analysis protein expression of survivin. The data are the means ± standard deviation (SD) of three independent experiments. *P<0.05, C3 + C2, C1, C0 vs. normal control; ^#^P<0.05, C3 + C2, C1 vs. C0; ^△^P<0.05, C3 + C2 vs. C1.

Immunocytochemical staining indicated that survivin-positive PBMCs were significantly upregulated in patients with good collateral formation, including plasma and nuclear regions (Fig. 1). Similarly, RT-PCR and western blotting indicated that the mRNA and protein expression of survivin in PBMCs was significantly higher in patients with good collateral circulation (C2 + C3) than that in patients with no collateral flow (C0) (P<0.05), although no significant differences were observed between C2 and C3 patients (P>0.05) (Fig. 2).

### CD4, CD8, CD44, VEGF and ICAM-1 protein expression in human PBMCs during coronary collateral formation

**Figure 3:**
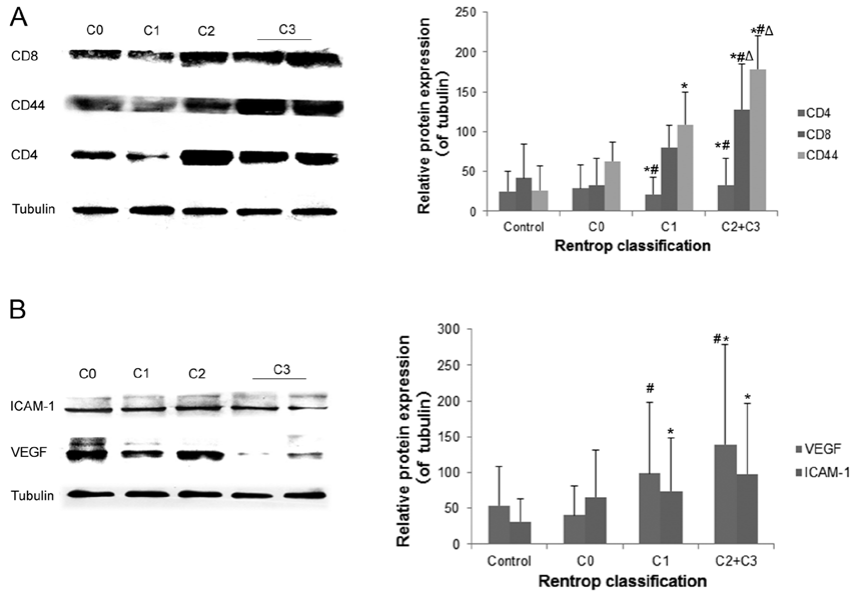
CD4, CD8, CD44, vascular endothelial growth factor (VEGF) and intercellular adhesion molecule-1 (ICAM-1) expression in human peripheral blood mononuclear cells (PBMCs) during coronary collateral formation. CD4, CD8, CD44, VEGF and ICAM-1 expression was determined by western blotting. Tubulin was used as an internal control. (A) CD4, CD8 and CD44 protein expression and (B) VEGF and ICAM-1 protein expression. The data are the means ± SD of three independent experiments. ^*^P<0.05, C3 + C2, C1, C0 vs. normal control; ^#^P<0.05, C3 + C2, C1 vs. C0; ^△^P<0.05, C3 + C2 vs. C1.

Western blot analysis revealed that CD8 and CD44 expression in PBMCs was significantly higher in patients with good collateral formation (C3 + C2) than in patients with poor collateral formation (C1), no collateral formation (C0), and normal controls (all P<0.05) (Fig. 3). CD4 and VEGF expression were also higher in patients with good collateral circulation than in those with no collateral flow (C0) and normal control patients (all P<0.05). However, this result was non-significant between patients with good collateral circulation (C3 + C2) and poor collateral circulation (C1) (P>0.05). ICAM-1 expression was higher in patients with collateral formation than that in normal control patients (all P<0.05). However, this result was non-significant among good collateral (C3 + C2), poor collateral (C1), and no collateral (C0) groups (P>0.05).

### Flow cytometric analysis of survivin single- and double-positives in human PBMCs

**Figure 4:**
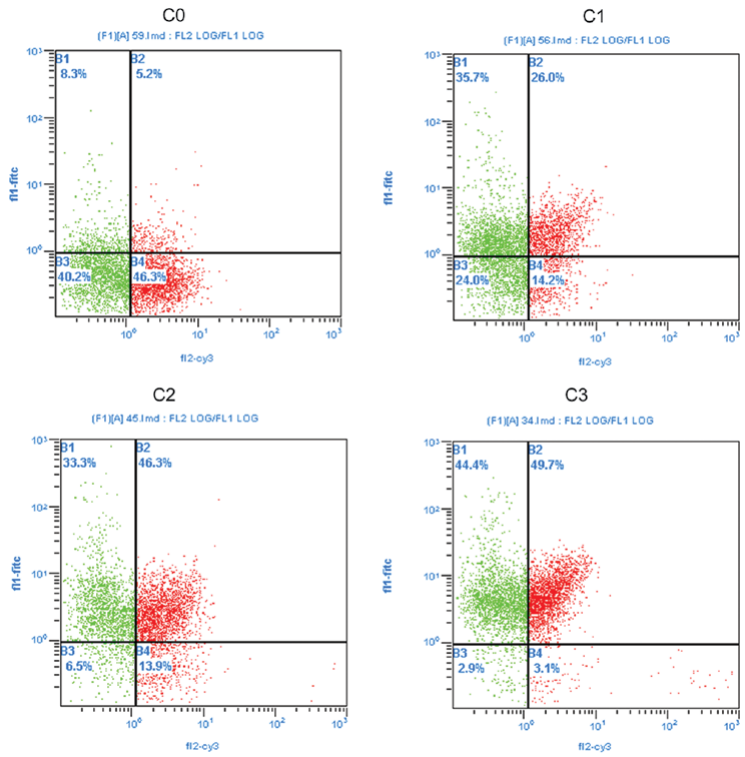
Flow cytometric analysis for survivin (FITC, green) + vascular endothelial growth factor (VEGF) (Cy3, red) double-positive in human peripheral blood mononuclear cells (PBMCs) during coronary collateral formation.

Flow cytometric analysis revealed that the percentages of survivin and CD44 single-positive PMBCs, survivin and CD8 double-positive (survivin + CD8), survivin and VEGF double-positive (survivin + VEGF), and survivin and ICAM-1 double-positive (survivin + ICAM-1) PBMCs were significantly higher in patients with good collateral formation than those in the normal controls and those with no collateral formation (C0) (all P<0.05) (Fig. 4 and Table II). Percentages of survivin + CD8 and survivin + VEGF double-positive PBMCs were significantly higher in patients with good collateral formation than those in the normal control and with no collateral formation (C0) (all P<0.05).

### Postmortem angiography in rat models and PBMC survivin expression correlation

**Figure 5:**
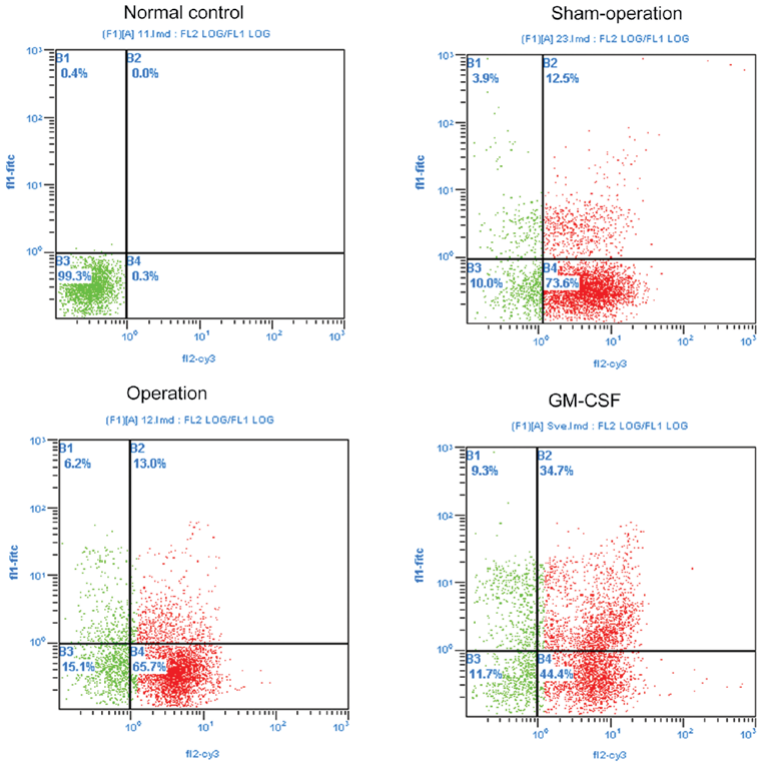
Flow cytometric analysis of survivin (FITC, green) + vascular endothelial growth factor (VEGF) (Cy3, red) double-positive in rat model of hind limb ischemia.

**Figure 6:**
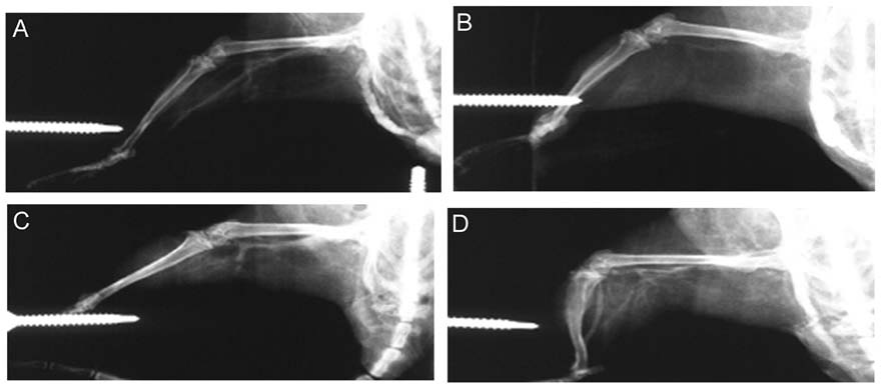
Collateral artery growth in postmortem angiography in rat model of hind limb ischemia. Angiograms in the (A) normal control and (B) operation groups revealed very poor collateral formation, whereas good collateral formation was observed in the (C) granulocye macrophage colony-stimulating factor (GM-CSF) treatment groups at 28 days after operation and (D) GM-CSF treatment groups at 42 days after operation, where collateral was most apparent.

In rat models, macroscopic collateral arteries were visible after right femoral artery ligation (operation and GM-CSF treatment groups) (Fig. 6). Percentages of survivin and CD44 single-positive PMBCs, and survivin + CD8, + VEGF, and + ICAM-1 double-positive PBMCs were observed significantly higher in the GM-CSF treatment group as compared to those observed in the remaining groups (all P<0.05). Percentages of survivin single-positive PMBCs, and survivin and CD8, VEGF and ICAM-1 double-positive PBMCs were significantly higher in the operation group than those in the sham-operation and normal control groups (all P<0.05) (Fig. 5 and Table III). Furthermore, in specimens with higher survivin levels, significantly larger and more visible collateral vessels were consistently observed.

## Discussion

Increased survivin expression in PBMCs was associated with greater coronary collateral formation in CTO patients. Moreover, the flow cytometric analysis revealed that the percentage of survivin-positive PBMCs was significantly higher in patients with good collateral formation than that in patients with poor collateral formation. These findings were confirmed in a rat model of hind limb ischemia, indicating that GM-CSF treatment after femoral artery ligation is capable of significantly promoting collateral formation. Notably, survivin, CD4, CD44 single-positive PBMCs as well as survivin + CD8, + VEGF, and + ICAM-1 double-positive PBMCs were increased, consistent with findings in samples from human cells. While survivin levels have previously been associated with CAD (4), this novel finding demonstrates that the survivin expression signature of PBMCs may increase collateral formation, potentially providing a valuable prognostic indicator in CAD patients, particularly those with coronary CTO. Furthermore, these observations are potentially useful in developing revascularization strategies.

The role of CD4^+^, CD8^+^ and CD44^+^ T lymphocytes in arteriogenesis and collateral development is well documented. Accumulating evidence has indicated that survivin is expressed in normal adult cells, particularly in primitive hematopoietic cells, T lymphocytes, polymorphonuclear neutrophils, and vascular endothelial cells, where it is important in cell proliferation, regulation, and survival (20). CD4^+^ T cells control the arteriogenic response to acute hind limb ischemia, in part by recruiting macrophages to the site of active collateral artery formation and triggering the development of collateral arteries through arteriogenic cytokine synthesis (21). During collateral formation, CD8^+^ T cells contribute to the early phases of collateral development. Following femoral artery ligation, CD8^+^ T cells infiltrate the site of collateral vessel growth and recruit CD4^+^ mononuclear cells by expressing IL-16 (21). Notably, CD44 deficiency has been reported to impede arteriogenesis (22), and increased CD44 expression on isolated monocytes is increased in patients with good collateralization (23). Confirming these findings, Arslan *et al* (24) reported a significant association between increased circulating CD14^+^ + CD16^‒^ monocyte levels and good coronary collateral development. Those findings are consistent with those of the present findings showing that CD4-, CD8- and CD44-positive T cells play a crucial role in collateral formation.

Expression of ICAM-1 and/or VEGF is critical to the growth of coronary collaterals (24,25). As shear stress upregulates the expression of endothelial cell adhesion receptors such as ICAM-1 and vascular cell adhesion molecule-1 (26,27), monocyte adhesion to shear stress-activated endothelium is important in arteriogenesis and in collateral artery growth (28). Hoefer *et al* (29) demonstrated that ICAM-1-mediated monocyte adhesion via ICAM-1/Mac-1 interaction to the endothelium of collateral arteries is essential in these processes by treating *in vivo* subjects with monoclonal antibodies against ICAM-1, which totally abolished the stimulatory effect of MCP-1 on collateral artery growth (30). Similarly, Rivard *et al* (31) reported that a reduced VEGF expression in diabetic mice resulted in impairment of new blood vessel formation, although cytokine supplementation using intramuscular adeno-VEGF gene transfer restored neovascularization (25). Consistent with the present study, ICAM-1 and VEGF are essential in many vascularization processes, including collateral formation. In particular, the present study demonstrates that expression of VEGF and ICAM-1 was elevated in patients with collateral formation. Furthermore, PBMCs in patients with elevated collateral evidenced distinctly higher rates of survivin single-positive as well as survivin + VEGF and + ICAM-1 double-positive cells, indicating that the mechanism of coronary collateral formation may be, in part, associated with high survivin, VEGF, ICAM-1 expression in PBMCs.

However, it has been shown that VEGF exerts anti-apoptotic effects during angiogenesis that may be mediated by the induced survivin expression in endothelial cells (31). Although these findings seem to limit the practical implementation of survivin upregulation, the importance of survivin during vascularization processes, including collateral formation is confirmed. However, the clinical relationships between compounds, such as VEGF and survivin, in human PMBCs merits further investigation prior to development of clinical strategies. It is important to consider also that, this study may be limited by the relatively small study group and use of a hind limb ischemia rat model as compared to the coronary collateral formation model due to the insufficiently small size of the rat coronary artery for contrast-based angiography. Furthermore, the rat hind limb model was selected for comparison with human coronary collateral formation as it has been reported that the femoral artery ligation rat model is simple and accurate for use in angiographic studies, whereas coronary artery ligation in rats is extremely difficult and less accurate (32,33). Thus, the present model has been demonstrated to produce low animal mortality and high success rates in numerous investigations of coronary CTO in the literature (32,33). Despite the use of this established model, any error occurring may be due to the discrepancies between the hind limb ischemia and coronary collateral rat models, which should be considered in the analysis of these results and design of future clinical studies.

In conclusion, in PMBCs from clinical coronary CTO patients and a rat model of hind limb ischemia, an elevated survivin expression in PBMCs, particularly of survivin and CD8, VEGF, ICAM-1 double-positive PBMCs were associated with good collateral formation. Thus, assessment of survivin level in the peripheral blood may provide a viable clinical alternative for CAD assessment. Furthermore, there may be potential applications of these findings in revascularization strategies for treating CAD patients that may undergo ischemic events due to disease or bypass surgery.

## Figures and Tables

**Table I:** Baseline clinical and demographic characteristics for CTO patients and normal controls.

Variables	Normal control (n=18)	Rentrop classification^a^			Total patients
		C0 (n=12)	C1 (n=18)	C2 + C3 (n=16)	(C0 – C3) (n=46)
Age (year)	58.0±10.0	58.8±8.6	61.5±9.4	64.1±8.5	60.1±8.5
Gender (M:F)	10/8	7/5	9/9	9/7	25/21
LAD	0	54.3±38.2	84.6±19.5	88.9±23.5	76.8±28.4
LCX	0	24.6±36.4	48.6±36.1	52.6±40.1	46.2±38.1
RCA	0	22.2±32.4	43.6±34.7	45.9±45.9	40.5±38.3
TC (mmol/l)	4.96±0.11	4.77±0.72	4.65±0.76	4.96±0.71	4.80±0.78
TG (mmol/l)	1.57±0.10	1.74±0.91	1.89±0.81	2.06±0.71	1.92±0.86
LDL (mmol/l)	2.86±0.12	2.50±0.60	2.26±0.64	2.36±0.55	2.38±0.72
HDL (mmol/l)	1.39±0.10	1.45±0.19	1.40±0.22	1.43±0.27	1.43±0.33

a Rentrop classification: C0, no collateral; C1, poor collateral; C2 + C3, good collateral. CTO, chronic total occlusion; LAD, left anterior descending; LCX, left circumflex artery; RCA, right coronary artery; TC, total cholesterol; TG, triglyceride; LDL, low-density lipoprotein; HDL, high-density lipoprotein.

**Table II:** Flow cytometric analysis of survivin and CD44 single-positive, and survivin and CD4, CD8, VEGF and ICAM-1 double-positive in human PBMCs during coronary collateral formation.

Rentrop classification	Survivin %	Sur + CD4 %	Sur + CD8 %	CD44 %	Sur + VEGF %	Sur + ICAM-1 %
Normal control	6.2±2.5	56.1±6.2	5.1±2.2	10.6±4.1	9.0±3.9	4.4±2.1
C0	21.6±10.8^a^	38.4±6.8^a^	4.4±0.9	17.5±5.7	19.5±4.6^a^	5.0±1.4
C1	19.7±15.9	37.2±8.6^a^	3.8±1.5	31.1±8.3^a^,^b^	34.7±8.6^a^,^b^	3.0±1.1^b^
C2 ± C3	45.0±16.2^a^–^c^	31.9±7.4^a^	36.5±5.1^a^–^c^	65.4±8.2^a^–^c^	54.6±6.2^a^–^c^	21.4±5.4^a^–^c^

Data are the means ± SD of three independent experiments.

a P<0.05, C3 + C2, C1, C0 vs. normal control;

b P<0.05, C3 + C2, C1 vs. C0;

c P<0.05, C3 + C2 vs. C1. PBMCs, peripheral blood mononuclear cells; SD, standard deviation; VEGF, vascular endothelial growth factor; ICAM-1, intercellular adhesion molecule-1.

**Table III:** Flow cytometric analysis for survivin and CD44 single-positive, and survivin and CD4, CD8, VEGF and ICAM-1 double-positive in rat model of hind limb ischemia.

Group	Survivin %	Sur + CD4 %	Sur + CD8 %	CD44 %	Sur + VEGF %	Sur + ICAM-1 %
Normal control	6.3±2.3	55.2±6.4	5.3±2.1	10.0±4.2	7.8±3.7	4.8±2.2
Sham-operation	7.1±2.9	48.4±7.1	4.8±1.2	11.5±5.6	10.7±2.3	4.8±1.3
Operation	16.9±6.1^a^,^b^	35.8±7.7^a^,^b^	5.9±5.3	30.1±7.5^a^,^b^	25.1±7.5^a^,^b^	3.3±1.1
GM-CSF	40.5±13.1^a^–^c^	42.5±7.4^a^	33.7±7.4^a^–^c^	50.5±7.0^a^–^c^	52.0±9.0^a^–^c^	20.5±5.8^a^–^c^

Data are the means ± SD of three independent experiments.

a P<0.05, GM-CSF, operation, sham-operation vs. normal control;

b P<0.05, GM-CSF, operation vs. sham-operation;

c P<0.05, GM-CSF vs. operation. GM-CSF, granulocyte macrophage colony-stimulating factor; VEGF, vascular endothelial growth factor; ICAM-1, intercellular adhesion molecule-1.
